# Physical Distancing Measures and Walking Activity in Middle-aged and Older Residents in Changsha, China, During the COVID-19 Epidemic Period: Longitudinal Observational Study

**DOI:** 10.2196/21632

**Published:** 2020-10-26

**Authors:** Yilun Wang, Yuqing Zhang, Kim Bennell, Daniel Kenta White, Jie Wei, Ziying Wu, Hongyi He, Shaohui Liu, Xianghang Luo, Shuo Hu, Chao Zeng, Guanghua Lei

**Affiliations:** 1 Department of Orthopaedics Xiangya Hospital Central South University Changsha China; 2 Division of Rheumatology, Allergy, and Immunology Department of Medicine Massachusetts General Hospital Boston, MA United States; 3 The Mongan Institute Massachusetts General Hospital Harvard Medical School Boston, MA United States; 4 Centre for Health, Exercise and Sports Medicine The University of Melbourne Melbourne Australia; 5 Department of Physical Therapy University of Delaware Newark, DE United States; 6 Health Management Center Xiangya Hospital Central South University Changsha China; 7 Department of Endocrinology, Endocrinology Research Center Xiangya Hospital Central South University Changsha China; 8 National Clinical Research Center of Geriatric Disorders Xiangya Hospital Central South University Changsha China; 9 Department of Nuclear Medicine Xiangya Hospital Central South University Changsha China; 10 Hunan Key Laboratory of Joint Degeneration and Injury Changsha China

**Keywords:** COVID-19, pandemic, physical distancing, steps, walking activity

## Abstract

**Background:**

Physical distancing measures taken to contain COVID-19 transmission may substantially reduce physical activity levels and cause individuals to adopt a more sedentary lifestyle.

**Objective:**

The objective of this study is to determine if there was any change in daily steps, an important component of daily physical activity, and examine risk factors for frequent low daily steps during the COVID-19 epidemic.

**Methods:**

We used data collected from the Step Study, a population-based longitudinal study of walking activity among residents aged ≥40 years in Changsha, China. Daily steps were collected via a smartphone linked to WeChat, a social networking platform. We plotted mean daily steps and the prevalence of low daily steps (≤1500 steps/day) 30 days before (reference period) and 30 days after (epidemic period) January 21, 2020 (date of the first COVID-19 case diagnosed in Changsha), and compared it with the same corresponding period from 2019. We examined the association of risk factors with the prevalence of frequent low daily steps (≤1500 steps/day for ≥14 days) using logistic regression.

**Results:**

Among 3544 participants (mean age 51.6 years; n=1226 females, 34.6%), mean daily steps dropped from 8097 to 5440 and the prevalence of low daily steps increased from 3% (2287/76,136 person-day) to 18.5% (12,951/70,183 person-day) during the reference and epidemic periods, respectively. No such phenomenon was observed during the corresponding period in 2019. Older age (*P* for interaction=.001) and female sex (*P* for interaction<.001) were both associated with a higher prevalence of frequent low daily steps and were more pronounced during the epidemic period. More education was associated with a lower prevalence of frequent low daily steps during the reference period but not the epidemic period (*P* for interaction=.34). Body mass index or comorbidity were not associated with frequent low daily steps during either period.

**Conclusions:**

Daily steps of Changsha residents aged ≥40 years dropped significantly during the COVID-19 period, especially among older adults and females. Although successful physical distancing, measured by the rapid downward trend in daily step counts of residents, played a critical role in the containment of the COVID-19 epidemic, our findings of an increase in the prevalence of frequent low daily steps raise concerns about unintended effects on physical activity.

## Introduction

COVID-19 has caused morbidity and mortality worldwide [1]. To control this highly contagious infectious disease, many countries have implemented “physical distancing” and “shelter-in-place” measures to contain COVID-19 transmission [2,3]. However, such measures may substantially reduce physical activity levels and cause individuals to adopt a more sedentary lifestyle [4].

There is ample evidence that regular physical activity is crucial for health, with physical inactivity characterized as the fourth leading cause of global mortality [5]. Results from the National Health and Nutrition Examination Survey (NHANES) and the Women’s Health Study both showed that a lower number of daily steps, which is an important component of daily physical activity, was significantly associated with higher all-cause mortality [6,7]. Studies also demonstrated that individuals who walked less than 1500 daily steps for 14 days were at a higher risk of muscle mass loss and low insulin sensitivity [8,9]. To date, several studies have reported changes in daily steps of residents in areas affected by COVID-19 [10-14]; however, none of these studies specifically described the prevalence of frequent low daily steps (≤1500 steps/day for ≥14 days over one month) [8,9], a strong predictor of poor health outcomes, and examined its risk factors.

Using data collected from the Step Study, a longitudinal study conducted among the urban residents of Changsha, China, we therefore described the trends of daily steps around the COVID-19 epidemic period in 2020 and compared them with that of a similar period one year earlier. We examined the relationships between several sociodemographic factors, anthropometric factors, and comorbidity and the prevalence of frequent low daily steps.

## Methods

### Study Population

The Step Study (registration number: ChiCTR1800017977) is a population-based longitudinal study initiated in September 2018 in Changsha, China. The aims of the Step Study were to describe patterns of walking activity, factors related to walking activity, and its sequelae among the community-living population. Participants in the Step Study comprised individuals who had their annual physical checkup at Xiangya Hospital of Central South University in Changsha, China [15,16]. To be eligible for the Step Study, participants had to meet the following criteria: (1) be a resident of Changsha; (2) be aged ≥40 years; (3) own a personal smartphone and have a WeChat account; and (4) be willing and able to give consent. Individuals with severe mental illness were ineligible. During each annual physical checkup, participants were queried about their sociodemographic and lifestyle factors, comorbidities, health-related symptoms and signs (eg, joint pain), and ability to perform daily activities. They also received clinical examinations and laboratory tests, including physical function tests (eg, lower limb muscle strength measurements).

For each participant, walking activity measured as daily step counts was collected through a smartphone linked to WeChat. WeChat is a multipurpose social network platform (Tencent Inc) with approximately 1.1 billion monthly active users in China [17]. One of its apps can extract daily step count information from the accelerometer sensor in a smartphone. Thus, if a participant is a WeChat user and wears a smartphone, his/her step counts can be captured by WeChat’s app.

### Study Outcome

The outcome variable was daily step counts recorded by the accelerometer sensor in the smartphone and extracted by WeChat. To be eligible for the current analysis of daily step counts, we required participants to wear their smartphone for ≥10 hours on a given day, a standard convention for measuring daily walking [18,19]. The smartphone wear time was calculated as the difference between the times of the first recorded step count and the last recorded step count each day. This algorithm was used in the Activity Inequality Project to calculate daily step counts for more than 700,000 individuals across 111 countries [19]. Participants with no valid daily step count were excluded in the current analysis. We defined a low daily step count as ≤1500 steps/day [8,9]. If a participant had ≥14 days of low daily step counts over a 30-day period, we considered that person as having experienced frequent low daily steps [8,9].

We conducted two validation studies to assess the accuracy of daily step counts collected from WeChat. To determine the accuracy of steps measured at various walking speeds by iOS and Android devices, we visually counted steps from 14 subjects walking on a treadmill at 4.8, 6.4, 8.0, and 9.6 km/h while subjects held/wore smartphones in different positions (ie, pants pocket, hand, and arm). These methods are consistent with previous studies [20-22]. We found step count accuracy to be high, with intraclass correlation coefficients (ICCs) ranging from 0.64 to 0.99. Second, we assessed the accuracy of step counts extracted from WeChat in free-living conditions. Specifically, 36 participants from the Step Study were instructed to wear both a Fitbit Charge 3 (Fitbit Inc), as the criterion measure [23], and their personal smartphone for 7 consecutive days [24]. The results also demonstrated a moderate to high agreement on step counts, with ICCs ranging from 0.67 to 0.81. Detailed information from these validation studies is shown in Multimedia Appendix 1.

### Study Exposures

Information on age, sex, educational level, height, weight, and comorbidity were obtained from the annual physical checkup visit. Body mass index was calculated. The modified Charlson Comorbidity Index (CCI) was computed based on self-reported comorbidities [25].

### Statistical Analyses

On January 22, 2020, one day after the first case of COVID-19 was diagnosed in Changsha (January 21, 2020, ie, the index day) the municipal government issued an emergency notice to implement physical distancing measures (ie, staying at home, closing schools, working from home if possible, travelling only when necessary, and cancelling mass gatherings) [26,27]. In this analysis, we defined the time interval from January 22 to February 20, 2020 (30 days after the index date), as the COVID-19 epidemic period, and the time interval from December 22, 2019, to January 20, 2020 (30 days before the index date), as the reference period. Since the COVID-19 epidemic occurred around the holiday season of Chinese New Year, for the purpose of comparison we also used data collected between January 2 and March 3, 2019, which corresponded to the same Chinese lunar calendar period. Details of the selection of study periods are shown in Multimedia Appendix 2.

First, we plotted the mean daily steps from December 22, 2019, to February 20, 2020 (around COVID-19 epidemic period), and mean daily steps from January 2 to March 3, 2019 (historic comparison period), respectively, using a 3-day moving average smooth method [28]. Second, we used the same approach to plot the prevalence of low daily steps (≤1500 steps/day) for the corresponding periods. We calculated the mean difference (MD) and its 95% confidence interval for daily steps using Generalized Estimating Equations (GEE) between the epidemic period and reference period in 2020, and the corresponding periods in 2019, respectively. Specifically, we included each qualified daily step count into the GEE model using the PROC GENMOD procedure in SAS (Version 9.4; SAS Institute) with identity links to calculate the MD and its 95% CI between the epidemic and reference periods. We added the REPEATED statement to account for correlation of the repeated measurements of individuals’ daily step counts [29].

Similarly, prevalence ratios (PR) were calculated for the prevalence of low daily steps (≤1500 steps/day) between the two comparative time periods in 2019 and 2020, respectively [30]. Finally, we estimated the prevalence of frequent low daily steps (≤1500 steps/day for ≥14 days over 30 days) during epidemic and reference periods and examined whether age, sex, BMI, educational level, and comorbidity were associated with the prevalence of frequent low daily steps using logistic regression. We tested the additive effect measure of modification of physical distancing with each of the risk factors mentioned above by adding an interaction term in the regression model [31].

All *P* values for interaction were two-sided and *P* for interaction<.05 was considered statistically significant for all tests. All statistical analyses were conducted using SAS (Version 9.4).

### Ethical Considerations

This study was approved by the Ethics Committee of Xiangya Hospital, Central South University (#201806910), and written informed consent was obtained from study participants.

## Results

A total of 7262 Changsha residents aged ≥40 years had a physical checkup at the study center between September 2018 and January 2020, and 4145 of them (57.1%) had a WeChat account and agreed to participate in the Step Study. Of these, 3544 with at least one valid daily step count during the study period were included in the analysis (Table 1). The mean age of participants was 51.6 (SD 8.9) years, 1226 (34.6%) participants were females, and the mean BMI of participants was 24.0 (SD 4.3) kg/m^2^. Overall, 2616 (73.8%) participants were Android users, 765 (21.6%) were iOS users, and 163 (4.6%) participants’ phone types were unknown.

**Table 1:** Baseline characteristics of included participants.

Characteristics		Total sample (N=3544)	Males (n=2318)	Females (n=1226)
Age (years), mean (SD)		51.6 (8.9)	51.6 (8.8)	51.5 (9.0)
**Age (years), n (%)**				
	40-49	1733 (48.9)	1117 (48.2)	616 (50.2)
	50-59	1190 (33.6)	818 (35.3)	372 (30.3)
	60-70	452 (12.7)	271 (11.7)	181 (14.8)
	≥70	169 (4.8)	112 (4.8)	57 (4.7)
BMI (kg/m^2^), mean (SD)^a^		24.0 (4.3)	24.8 (4.2)	22.5 (4.3)
**BMI (kg/m^2^), n (%)^b^**				
	<25	1908 (59.6)	1043 (49.2)	865 (80.0)
	≥25	1294 (40.4)	1075 (50.8)	219 (20.0)
**Education, n (%)^b^**				
	High school or below	564 (22.1)	328 (19.7)	236 (26.5)
	Junior college	621 (24.3)	372 (22.4)	249 (27.9)
	University or above	1369 (53.6)	962 (57.9)	407 (45.6)
**Charlson Comorbidity Index, n (%)**				
	0	2759 (77.8)	1789 (77.2)	970 (79.1)
	≥1	785 (22.2)	529 (22.8)	256 (20.9)

^a^N=3202.

^b^N=2554.

**Figure 1:**
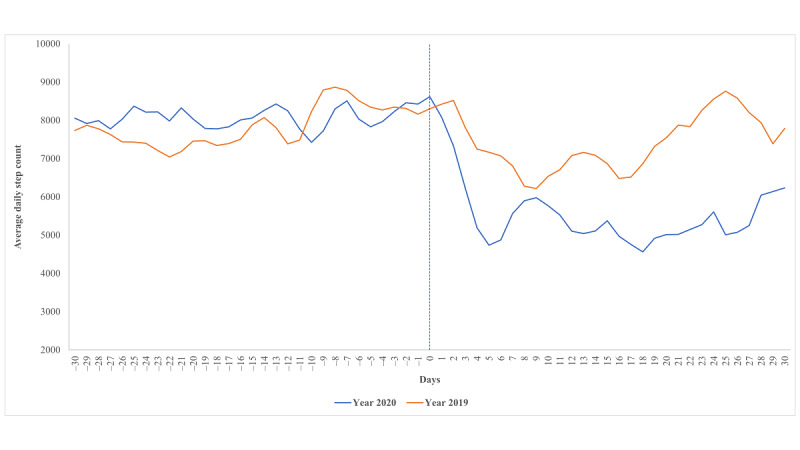
Average daily step count around the Chinese Lunar New Year period among participants in 2019 and 2020. Day 0 represents the index date; in 2020, this represents the date of the first COVID-19 case diagnosed in Changsha.

As shown in Figure 1 and Table 2, daily steps (mean 8097 steps/day, range 6942-9153 steps/day) during the reference period (30 days prior to the COVID-19 epidemic in 2020) were similar to that during the corresponding period in 2019 (mean 7872 steps/day, range 6649-8912 steps/day). However, the daily steps decreased substantially after implementing physical distancing measures, from 8624 steps/day on the day before the index day to 4121 steps/day on Day 4 after the index day; this trend continued during the rest of the epidemic period. Compared with the reference period, the mean daily steps dropped by 2678 steps (95% CI 2582-2763). However, this trend was not observed during the corresponding period in 2019.

**Table 2:** Associations of time period with daily step count.

Time period	2019 daily step count		2020 daily step count	
	Mean (SD)	Mean difference (95% CI)^a^	Mean (SD)	Mean difference (95% CI)^a^
Reference	7872 (4842)	0 (reference)	8097 (4793)	0 (reference)
Epidemic	7472 (4979)	–413 (–501 to –325)	5440 (4571)	–2672 (–2763 to –2582)

^a^Mean differences were adjusted for age and sex.

**Figure 2:**
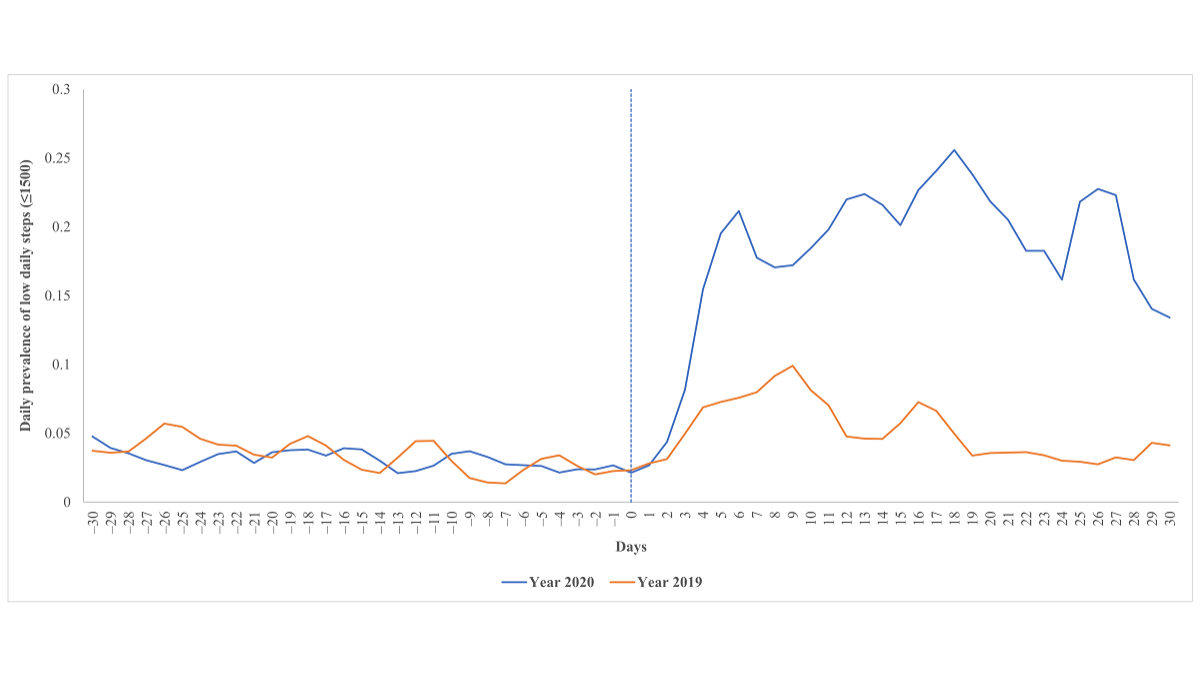
Prevalence of low daily step count (≤1500 steps/day) around the Chinese Lunar New Year period among participants in 2019 and 2020. Day 0 represents the index date; in 2020, this represents the date of the first COVID-19 case diagnosed in Changsha.

Figure 2 and Table 3 present the prevalence of low daily steps (≤1500 steps/day) in 2019 and 2020. The prevalence of low daily steps was similar during the reference period in 2020 (3.0%, 2287/76,136 person-day) and the corresponding period in 2019 (3.3%, 1006/30,647 person-day). In contrast, the prevalence of low daily steps increased substantially after implementing physical distancing measures, from 2.0% (53/2693) on the day prior to the index date to 25.5% (639/2505) on Day 4 after the index date; this trend continued during the rest of the follow-up period. Compared with the reference period, the PR of low daily steps was 6.2 (95% CI 5.8-6.7). However, no such trend was observed during the entire historic comparison period in 2019.

**Table 3:** Associations of time period with prevalence of low daily steps (≤1500 steps/day).

Time period	2019 low daily steps			2020 low daily steps	
	Person-day, n (%)	Prevalence ratio (95% CI)^a^	Person-day, n (%)		Prevalence ratio (95% CI)^a^
Reference	1006 (3.3)	1.0 (reference)	2287 (3.0)		1.0 (reference)
Epidemic	1488 (5.1)	1.6 (1.4-1.7)	12,951 (18.5)		6.2 (5.8-6.7)

^a^Prevalence ratios were adjusted for age and sex.

As shown in Table 4, only 12 (0.4%) of 2879 participants had walked less than ≤1500 steps/day for 14 days or more (frequent low daily steps) during the 2020 reference period; however, the prevalence of frequent low daily steps increased to 7.4% (196/2655) during the COVID-19 epidemic period, after physical distancing measures were implemented. Older age (*P* for interaction=.001) and female sex (*P* for interaction<.001) were both associated with a higher prevalence of frequent low daily steps than their counterparts during the reference and COVID-19 epidemic periods, and such associations were more pronounced during the epidemic period. Participants with a university education or more had a lower prevalence of frequent low daily steps than those with only high school or less education during the reference period but this was not observed during the COVID-19 epidemic period. During the physical distancing period, no significant interaction between education and prevalence of frequent low daily steps was observed (*P* for interaction=.34). There was no apparent association of either BMI or CCI with the prevalence of frequent low daily steps during either period.

**Table 4:** Association of prevalence of frequent low daily steps cases (≤1500 steps/day for ≥14 days) and basic characteristics.

Characteristics		Reference period			Epidemic period			*P* values for interaction
		Participants, n	Cases, n (%)	PR^a^ (95% CI)^b^	Participants, n	Cases, n (%)	PR (95% CI)^b^	
Total		2879	12 (0.4)	N/A^c^	2655	196 (7.4)	N/A	N/A
**Age (years)**								.001
	40-49	1543	1 (0.1)	1.0 (ref)^d^	1470	84 (5.7)	1.0 (ref)	N/A
	50-59	957	8 (0.8)	9.9 (1.2-0.0)	871	69 (7.9)	1.7 (1.2-2.3)	N/A
	60-70	308	2 (0.7)	9.2 (0.8-102.6)	257	32 (12.5)	2.2 (1.5-3.4)	N/A
	≥70	71	1 (1.4)	21.0 (1.3-331.8)	57	11 (19.3)	3.0 (1.6-5.7)	N/A
**Sex**								<.001
	Males	1904	7 (0.4)	1.0 (ref)	1793	75 (4.2)	1.0 (ref)	N/A
	Females	975	5 (0.5)	2.2 (0.6-7.9)	862	121 (14.0)	3.4 (2.5-4.6)	N/A
**Body mass index**								.250
	<25	1557	7 (0.5)	1.0 (ref)	1415	109 (7.7)	1.0 (ref)	N/A
	≥25	1006	3 (0.3)	0.8 (0.2-3.2)	269	61 (6.2)	1.2 (0.9-1.7)	N/A
**Education**								.335
	High school or below	441	6 (1.4)	1.0 (ref)	389	45 (11.6)	1.0 (ref)	N/A
	Junior college	522	2 (0.4)	0.3 (0.1-1.7)	460	31 (6.7)	0.7 (0.4-1.0)	N/A
	University or above	1228	2 (0.2)	0.2 (0.0-1.1)	1134	90 (7.9)	1.0 (0.7-1.4)	N/A
**Charlson Comorbidity Index**								.345
	0	2294	9 (0.4)	1.0 (ref)	2115	150 (7.1)	1.0 (ref)	N/A
	≥1	585	3 (0.5)	0.9 (0.2-3.8)	540	46 (8.5)	1.1 (0.7-1.5)	N/A

^a^PR: prevalence ratio.

^b^Prevalence ratios were adjusted for age and sex.

^c^N/A: not applicable.

^d^Ref: reference.

## Discussion

### Principal Findings

Using objective data collected from the longitudinal Step Study, we found that daily steps among middle-aged and older residents in Changsha dropped rapidly and substantially (2672 fewer daily steps on average) after implementing physical distancing measures during the COVID-19 epidemic period. In addition, more than 7% (196/2655) of residents had walked ≤1500 steps/day for ≥14 days over the one-month epidemic period compared with 0.4% (12/2879) of residents in the month prior to the epidemic. The reduction of steps/day during the COVID-19 epidemic was more pronounced among older adults and females.

### Comparison With Previous Studies

To date, several studies have reported changes in daily steps during the COVID-19 epidemic [10-14]. One worldwide study based on a smartphone app (Argus) showed that mean daily steps in different regions decreased by 5.5% and 27.3% (287 and 1432 steps/day, respectively) within 10 and 30 days after the COVID-19 pandemic was declared [10]. Another study that used a wristwatch with an embedded accelerometer (Withings) demonstrated a marked decrease in daily steps (from 25% to 54%) following the official dates of home confinement in countries adopting a total lockdown [11]. Similar findings were also reported in other countries [12-14]. Our results demonstrated that such a change also occurred in the Chinese population. Furthermore, we examined daily steps within the same period in the previous year and observed no such change during this period, which enabled us to minimize the potential impact of the holiday season of Chinese New Year on daily steps.

In addition, we found that the effect of implementation of physical distancing measures on frequent low daily steps was more pronounced among older adults and females. Previous studies have examined associations between walking activities and sociodemographic factors, anthropometric factors, and comorbidity. The results from the NHANES report indicated that those of advancing age (OR 1.95, per 16.7-year increments) and female sex (OR 1.86) both had higher odds of walking less than 5000 steps/day [32]. Another study found that education was associated with increased walking activity, with one additional year of education associated with a 560 daily steps increase [33]. Our results corroborate these findings. However, after the implementation of physical distancing measures, the prevalence of frequent low daily steps among residents with university or above education was similar to those with high school or below education, suggesting both groups followed physical distancing measures and reduced their outdoor walking activities. Nevertheless, the magnitude of relative increase in the prevalence of frequent low daily steps during the COVID-19 epidemic appeared to be greater among residents with university or above education than among those with high school or below education, indicating the former are more likely to follow instructions and communicate effectively with health providers [33,34]. Previous studies also showed that both BMI and comorbidities (eg, hypertension and diabetes) were associated with fewer daily steps [6,7,35,36], but this was not the case in the present study, nor was any association modified by the implementation of physical distancing measures.

### Public Health Implications

Physical distancing measures play a critical role in containing COVID-19 transmission and monitoring population mobility data can provide evidence as to whether people are complying with these measures [37]; however, the impact of physical distancing on other aspects of daily life should not be overlooked. Recently, Hall and colleagues [4] commented that “The world is experiencing an extraordinary, life-altering challenge due to the COVID-19 pandemic. Many countries have become accustomed to a new normal – ‘social distancing’ and ‘shelter-in-place’ are now a part of everyday vernacular and life.” The authors warned that this health crisis has the potential to further impact and accelerate the pandemic of physical inactivity and sedentary behavior.

Our data showed that average daily steps among residents of Changsha dropped by more than 30% (from 8097 to 5440) during the COVID-19 epidemic period after implementation of physical distancing measures. These data raise concerns about the potential adverse effects of such measures on health and well-being. Previous studies have showed that higher daily steps are associated with better cardiometabolic profiles and lower all-cause mortality [6,7,38]. Other studies have also found that a decrease in daily steps of 2000 steps, irrespective of previous habitual step counts, increases the risk of insulin sensitivity and higher cardiovascular events [39,40]. Thus, our findings have important implications for public health recommendations and the prevention of other health crises during the COVID-19 pandemic. Furthermore, our data showed that the reduction in daily steps is much more common among older adults and females. In general, older adults and females are more likely to develop sedentary behaviors, placing them at greater risk of various diseases related to inactivity [32,41]; thus, the worsening trend of physical inactivity during the COVID-19 epidemic period compounds the risk of sequelae related to a sedentary lifestyle.

It is uncertain as to how long it will take to completely control the COVID-19 pandemic worldwide. If similar trends toward a sedentary lifestyle are seen in other countries, the avoidance of further sedentary lifestyle behaviors and promotion of regular physical activity during this time are an urgent global public health issue. It is also unknown whether the observed decline in daily walking is a temporary phenomenon that may revert back to baseline levels after the disease is under control; thus, further longitudinal studies are needed so that evidence-based strategies can be developed and implemented to encourage greater participation in regular physical activity.

### Strengths and Limitations

Several strengths of our study are noteworthy. First, we used data from a large population-based longitudinal study (Step Study), which allowed not only investigation of COVID-19–related changes in daily step counts from over 3500 residents, but also inclusion of a historic comparison period from the prior year to account for a secular trend in daily step counts. Second, the study took advantage of a social network platform (WeChat) to capture daily step count data via smartphone in real time among the community-living population. As a smartphone has become a daily necessity for most adults, this approach allowed long-term monitoring of daily step count trends. This contrasts to previous studies using wearable devices (accelerometers) that generally only collect step count data for a relatively short time (eg, a week or less) [42]. It can be challenging to extrapolate such short-term data to describe step count patterns over a long period owing to various potential confounders [42].

Potential limitations of our study also deserve comment. First, the WeChat app may underestimate daily step counts because some individuals may not always carry their smartphone with them [43], especially when participants were housebound. Thus, some light walking activities at home may not be captured. However, one previous study reported that the average wear time of smartphones among Chinese citizens was more than 13 hours during the day time, indicating that most walking activities should be captured by a smartphone [19]. Second, although our validation study demonstrated that daily step counts collected from iOS and Android devices both showed close agreement with actual step counts under controlled laboratory settings, previous studies found that iOS and Android devices have different accuracies in capturing the daily step count under free-living conditions [44]. This could have led to misclassification of daily step counts in this study. Third, participants in our study were slightly younger (51.6 versus 54.8 years) and more likely to be male (65.4% versus 51.2%) than those who are aged ≥40 years and live in Changsha, according to the latest census data in 2010. However, our results showed that the prevalence of frequent low daily steps increased more among women and older people, suggesting that the overall prevalence of frequent low steps among all residents in Changsha aged ≥40 years may be even higher than what was reported in our study. Fourth, participants in our study were urban residents in Changsha who came to Xiangya Hospital for their annual health checkup. The percentage of WeChat users among these individuals (57.1%) was higher than that of the population with the same age range in China (41.5%) [45]. Thus, our findings may not be generalizable to residents living in other parts of China, especially those living in rural areas. Finally, we were unable to capture the intensity of steps (eg, slow versus fast steps); however, total steps/day has recently been shown to be an important predictor of mortality independent of step intensity [6].

### Conclusions

Using data collected from a large population-based longitudinal study, we demonstrated that walking activity, indicated by daily step count, decreased rapidly and substantially during the COVID-19 epidemic period among middle-aged and older adult Chinese residents living in urban areas. These results suggest that appropriate strategies need to be taken to encourage residents to actively engage in regular physical activity while maintaining personal hygiene and physical distancing. They also call for further studies to evaluate whether the low levels of walking activity observed following the implementation physical distancing measures will be maintained and whether they will have significant adverse impacts on health outcomes.

## Appendix

Multimedia Appendix 1 Validation study.

Multimedia Appendix 2 Diagram of the study periods. Chinese New Year fell on January 25 in 2020 and February 5 in 2019.

## Abbreviations

CCI: Charlson Comorbidity Index
GEE: Generalized Estimating Equations
ICC: intraclass correlation coefficients
MD: mean difference
NHANES: National Health and Nutrition Examination Survey
PR: prevalence ratio
