# Supplementary material for: Physical Distancing Measures and Walking Activity in Middle-aged and Older Residents in Changsha, China, During the COVID-19 Epidemic Period: Longitudinal Observational Study
Source: J Med Internet Res. 2020 Oct 26;22(10):e21632. doi: 10.2196/21632 (PMC7592463; doi:10.2196/21632)
Supplement: Multimedia Appendix 1 [file jmir_v22i10e21632_app1.docx]

**Laboratory test**

Fourteen volunteers were recruited to participate in the laboratory test. The smartphones used in this test were iPhone 6 and HUAWEI mate 8 [1, 2], which were the market leader of iOS and Android devices in China 2016, respectively [3]. All smartphones have already installed the WeChat. Each participant was equipped with three sets of iPhone 6 and three sets of HUAWEI mate 8 - one of each at three standardized positions on the body (i.e., left pants pocket, left hand, and right arm) [4, 5]. Then, each participant walked on a treadmill at four speeds: 4.8, 6.4, 8.0 and 9.6 km/h [6]. Between each trial participants came to a full stop by standing on the side of the treadmill for a 2-minute washout period. Each trial was filmed using a camera aimed at the participants’ lower body, and recorded step counts were used as the criterion measure [7]. Visual counts and WeChat counts were compared, and intra-class correlation coefficients (ICCs) were calculated (**Table 1**). All analyses were conducted with SPSS version 21.0 (SPSS Inc., Chicago, IL, USA).

**Free-living test**

We recruited 36 participants from the Step Study with their personal smartphones to verify the validity of step counting of WeChat in a free-living condition. All participants were instructed to wear Fitbit Charge 3 (Fitbit Inc. San Francisco, CA USA), as the criterion measure [8], and their personal smartphones throughout waking hours for a period of seven consecutive days [9]. Fitbit Charge 3 was placed on the participant’s dominant wrist. They were instructed to wear the Fitbit Charge 3 at all times except during sleep, shower, and water activities, and were told that for a day to be considered valid, the Fitbit Charge 3 must be worn for at least 10 hours [10]. Meanwhile, participants were also told to carry their personal smartphones while maintaining normal living conditions. Since insufficient wear-time of smartphone may underestimate the daily step counts, we considered a daily step counts as a valid record from WeChat if the wear-time of smartphone was ≥10 hours [11]. The wear-time of smartphone was calculated from the time of the first step count data was recorded to that of the time of the last step count data was recorded in each day [11]. After completion of the free-living test, the agreement of step counts recoded on Fitbit and step counts extracted from WeChat (Raw counts/validated counts) were assessed using ICCs (**Table 2**). All analyses were performed with SPSS version 21.0.

**Table 1. Intraclass correlation coefficients of step counts measured by WeChat and by visual count according to wearing locations of smartphone or walking speeds (n=14)**

| **Location** | **Speed (km/h)** | **HUAWEI mate 8** | | **iPhone 6** | |
| --- | --- | --- | --- | --- | --- |
|  |  | **ICCs** | **95% CI** | **ICCs** | **95% CI** |
| Left pants pocket | 4.8 | 0.98 | 0.93 to 0.99 | 0.80 | 0.48 to 0.93 |
|  | 6.4 | 0.99 | 0.97 to 1.00 | 0.95 | 0.86 to 0.99 |
|  | 8.0 | 0.98 | 0.93 to 0.99 | 0.92 | 0.58 to 0.98 |
|  | 9.6 | 0.95 | 0.87 to 0.99 | 0.92 | 0.76 to 0.97 |
| Left hand | 4.8 | 0.64 | 0.20 to 0.87 | 0.91 | 0.51 to 0.97 |
|  | 6.4 | 0.80 | 0.50 to 0.93 | 0.96 | 0.88 to 0.99 |
|  | 8.0 | 0.87 | 0.65 to 0.96 | 0.91 | 0.75 to 0.97 |
|  | 9.6 | 0.92 | 0.41 to 0.98 | 0.95 | 0.78 to 0.98 |
| Right arm | 4.8 | 0.83 | 0.54 to 0.94 | 0.91 | 0.60 to 0.97 |
|  | 6.4 | 0.94 | 0.83 to 0.98 | 0.97 | 0.90 to 0.99 |
|  | 8.0 | 0.90 | 0.56 to 0.97 | 0.97 | 0.91 to 0.99 |
|  | 9.6 | 0.93 | 0.67 to 0.98 | 0.95 | 0.83 to 0.98 |

ICCs, Intraclass correlation coefficients; CI, confidence interval.

**Table 2. Intraclass correlation coefficients of step counts measured by WeChat and by Fitbit Charge 3 in a free-living condition (n=36)**

|  | **Raw counts** | | **Validated counts^#^** | |
| --- | --- | --- | --- | --- |
|  | **ICCs** | **95% CI** | **ICCs** | **95% CI** |
| Day 1^st^ | 0.79 | 0.11 to 0.93 | 0.81 | 0.19 to 0.94 |
| Day 2^nd^ | 0.73 | 0.12 to 0.90 | 0.79 | 0.11 to 0.93 |
| Day 3^rd^ | 0.67 | 0.05 to 0.87 | 0.71 | 0.15 to 0.89 |
| Day 4^th^ | 0.74 | 0.18 to 0.90 | 0.81 | 0.31 to 0.93 |
| Day 5^th^ | 0.65 | 0.26 to 0.83 | 0.67 | 0.33 to 0.84 |
| Day 6^th^ | 0.79 | 0.20 to 0.92 | 0.81 | 0.27 to 0.94 |
| Day 7^th^ | 0.79 | 0.14 to 0.93 | 0.80 | 0.21 to 0.93 |
| 7-day average | 0.73 | 0.20 to 0.88 | 0.77 | 0.31 to 0.90 |

ICCs, Intraclass correlation coefficients; CI, confidence interval.

# Date were only accepted when the wear-time of participants’ smartphone ≥10 hours/day.

**References**

1. Case MA, Burwick HA, Volpp KG, Patel MS. Accuracy of smartphone applications and wearable devices for tracking physical activity data. JAMA. 2015 Feb 10;313(6):625-6. PMID: 25668268. doi: 10.1001/jama.2014.17841.

2. Hekler EB, Buman MP, Grieco L, Rosenberger M, Winter SJ, Haskell W, et al. Validation of Physical Activity Tracking via Android Smartphones Compared to ActiGraph Accelerometer: Laboratory-Based and Free-Living Validation Studies. JMIR Mhealth Uhealth. 2015 Apr 15;3(2):e36. PMID: 25881662. doi: 10.2196/mhealth.3505.

3. JIGUANG. 2017; Available from: <https://www.jiguang.cn/reports/41?winzoom=1>. Accessed 15 Mar 2017 [in Chinese].

4. Leong JY, Wong JE. Accuracy of three Android-based pedometer applications in laboratory and free-living settings. J Sports Sci. 2017 Jan;35(1):14-21. PMID: 26950687. doi: 10.1080/02640414.2016.1154592.

5. Duncan MJ, Wunderlich K, Zhao Y, Faulkner G. Walk this way: validity evidence of iphone health application step count in laboratory and free-living conditions. J Sports Sci. 2018 Aug;36(15):1695-704. PMID: 29179653. doi: 10.1080/02640414.2017.1409855.

6. Loprinzi PD, Smith B. Comparison Between Wrist-Worn and Waist-Worn Accelerometry. J Phys Act Health. 2017 Jul;14(7):539-45. PMID: 28290761. doi: 10.1123/jpah.2016-0211.

7. Orr K, Howe HS, Omran J, Smith KA, Palmateer TM, Ma AE, et al. Validity of smartphone pedometer applications. BMC Res Notes. 2015 Nov 30;8:733. PMID: 26621351. doi: 10.1186/s13104-015-1705-8.

8. Straiton N, Alharbi M, Bauman A, Neubeck L, Gullick J, Bhindi R, et al. The validity and reliability of consumer-grade activity trackers in older, community-dwelling adults: A systematic review. Maturitas. 2018 Jun;112:85-93. PMID: 29704922. doi: 10.1016/j.maturitas.2018.03.016.

9. Spence JC, Burgess J, Rodgers W, Murray T. Effect of pretesting on intentions and behaviour: a pedometer and walking intervention. Psychol Health. 2009 Sep;24(7):777-89. PMID: 20205026. doi: 10.1080/08870440801989938.

10. Troiano RP, Berrigan D, Dodd KW, Masse LC, Tilert T, McDowell M. Physical activity in the United States measured by accelerometer. Med Sci Sports Exerc. 2008 Jan;40(1):181-8. PMID: 18091006. doi: 10.1249/mss.0b013e31815a51b3.

11. Althoff T, Sosic R, Hicks JL, King AC, Delp SL, Leskovec J. Large-scale physical activity data reveal worldwide activity inequality. Nature. 2017 Jul 20;547(7663):336-9. PMID: 28693034. doi: 10.1038/nature23018.
