# Supplementary figures and images for: Physical Distancing Measures and Walking Activity in Middle-aged and Older Residents in Changsha, China, During the COVID-19 Epidemic Period: Longitudinal Observational Study
Source: J Med Internet Res. 2020 Oct 26;22(10):e21632. doi: 10.2196/21632 (PMC7592463; doi:10.2196/21632)

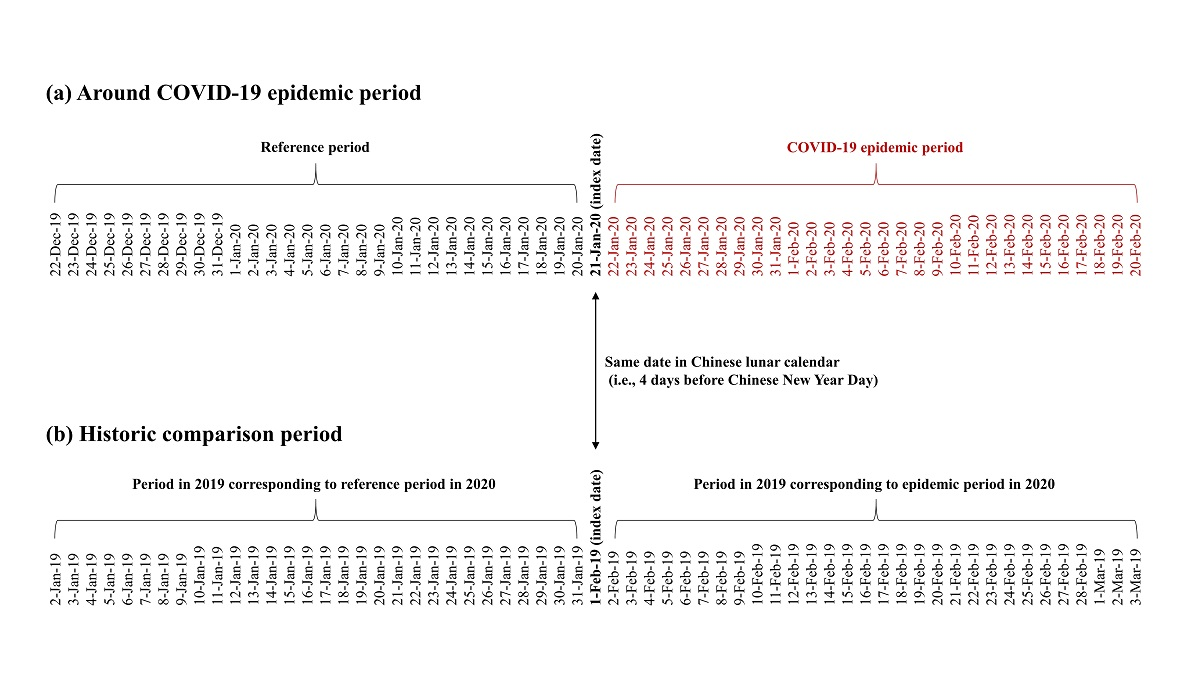

Supplement: Multimedia Appendix 2 [file jmir_v22i10e21632_app2.png]
